# *Polygalae Radix*: review of metabolites, pharmacological activities and toxicology

**DOI:** 10.3389/fphar.2024.1420853

**Published:** 2024-05-30

**Authors:** Meihua Liu, Xin Wang, Dejiang Gao

**Affiliations:** Research Center of Emotional Diseases, Shenyang Anning Hospital, Shenyang, China

**Keywords:** Polygalae Radix, metabolites, biological activity, immune system, toxicology

## Abstract

*Polygalae Radix*: is the dried root of *Polygala tenuifolia* Willd. or *Polygala sibirica* L., which has the effect of improving memory and cognitive function in traditional Chinese medicine. Modern pharmacological studies indicated that *Polygalae Radix* has rich pharmacological activities *in vitro* and *in vivo*, including protective effects on the nervous system, immune system, cardiovascular system and respiratory system, as well as antioxidant and antiepileptic pharmacological activities. Up to now, more than 160 metabolites from *Polygalae Radix* were identified, including triterpenoid saponins, xanthones, oligosaccharide esters and et al. The clinical practice of traditional Chinese medicine has proved that *Polygalae Radix* has a certain irritation to the throat, and a large or long-term use will stimulate the digestive tract, and the main toxic metabolite is saponins. Therefore, *Polygalae Radix* should be pr ocessed or used in combination with other Chinese herbal medicines to reduce the irritation to the throat and reduce gastrointestinal irritation. This article provides a review of the metabolites, pharmacological activity, and toxicology of *Polygalae Radix*. It also discusses the future research prospects and existing problems of *Polygalae Radix*, providing reference for further research on *Polygalae Radix*.

## 1 Introduction

There are more than 500 species of *Polygala* plants worldwide, with 39 species and 8 varieties in China, which are distributed throughout the country, mainly in the southwest and south China. There are 17 species and 2 varieties available for medicinal use in this genus (Editorial Committee of Flora of China, 1987; Zhang et al., 2002). *Polygalae Radix* (PR) recorded in Chinese Pharmacopoeia is the dry root of *Polygala tenuifolia* Willd. (*P. tenuifolia*) or *Polygala sibirica* L. (*P. sibirica*). It is called ‘Yuanzhi’ in Chinese and is a commonly used traditional Chinese medicine. PR is mainly distributed in China, South Korea, and Russia. In China, the main producing areas are Shanxi and Shaanxi provinces, which have the largest production and are traditionally considered to have the best quality of PR in these two provinces. In the northern of China such as Heilongjiang, Jilin, Liaoning, Gansu, Henan, Shandong, Anhui provinces also have a certain production of *Polygala tenuifolia* Willd., with a few being *Polygala sibirica* L. (Zhang et al., 2013; Liu et al., 2019).

**FIGURE 1:**
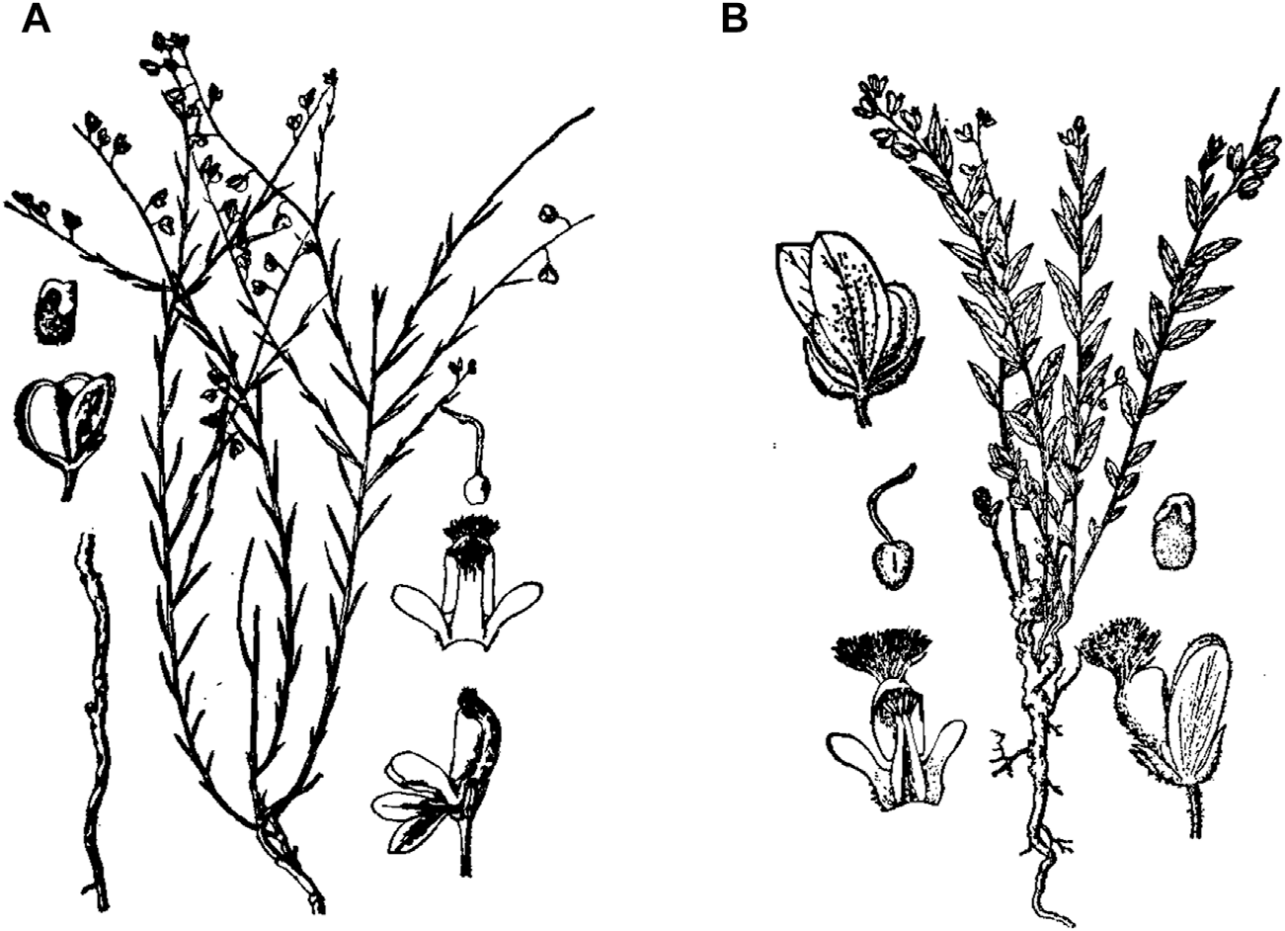
Plant diagram of *P. tenuifolia* and *P. sibirica*. **(A)** *P. tenuifolia*, **(B)** *P. sibirica*.

*P. tenuifolia* (Figure 1A) is a perennial herb with a plant height of 25–40 cm. The roots are cylindrical. The stem base is clustered, nearly glabrous. Leaves alternate, leaves linear or linear lanceolate, glabrous or very sparsely puberulent, subsessile. Racemes terminal. Capsule oblate ovoid, narrowly winged, glabrous. Seeds ovate, densely covered with white fine villi, flat black. The flowering period is from April to May, and the fruiting period is from June to July. The stem surface of *P. sibirica* (Figure 1B) is densely gray-brown puberulent. Leaf blade elliptic to oblong lanceolate. Racemes axillary exophytic or subterminal, pilose, less flowers. The capsule is nearly obovate, 5 mm in diameter, with narrow wings and marginal hairs. The important characteristics of *P. tenuifolia* and *P. sibirica* are shown in Table 1 (Institute of Botany, Chinese Academy of Sciences, 1972).

**TABLE 1:** The main characteristics of *P. tenuifolia* and *P. sibirica*.

Part of plant	*P. tenuifolia*	*P. sibirica*
Stem	Nearly glabrous	Dense grayish brown pubescent
Leaf	Linear or linear lanceolate; glabrous or extremely sparsely pubescent; near sessile	Oval to oblong lanceolate; both sides are pilose; with a short handle
Flowers	Racemes terminal	Racemes axillary exophytic or subterminal
Fruit	Fruit globose; unmarginal hairs	Nearly obcordate; jubate

*P. tenuifolia* and *P. sibirica* have the similar metabolites and can be used as medicine equally in Chinese medicine. Modern research has identified more than 160 metabolites from PR, mainly including saponins, xanthones, oligosaccharide esters, alkaloids and other effective metabolites (Chen et al., 2005; Wang et al., 2007). PR was first recorded in *Shennong’s Herbal Classic* which has a history of more than 2000 years in China. PR was mainly used to relieve cough and phlegm, benefiting intelligence and tranquilizing mind, detoxifying and detumescence, and tonifying and strengthening. It is commonly used in compatibility to improve patients’ cognition and memory ability, and it is also the main herb of the commonly used classic prescription ‘Kaixin Powder’ in TCM. At present, there are more than 870 kinds of traditional Chinese medicine prescriptions containing *Polygalae Radix* (Zhao et al., 2020), which are mainly used in the treatment of depression, anxiety, insomnia, Alzheimer’s disease, irregular menstruation, premature ovarian failure and other diseases (Yoo et al., 2014; Jin et al., 2015; Miao et al., 2017; Park et al., 2019).

At present, there are many studies on PR, but its mainly focus on its pharmacological activity on the nervous system, and has less research on its immune regulation, phlegm and cough and other pharmaceutical activity, and toxic side effects. In this paper, the metabolites and pharmacological activities of PR were systematically summarized, and the toxic side effects of PR were summarized and analyzed under limited research reports. The problems existing in the current PR research were further analyzed, and the direction of future research was discussed. This paper aims to provide a scientific basis for the development and utilization of PR medicinal value.

## 2 Metabolites

The chemical composition types of PR are rich. So far, more than 160 metabolites have been isolated and identified from *P. tenuifolia* and *P. sibirica*. There are mainly saponins, xanthones, oligosaccharide esters, alkaloids, phenylpropanoids, and lactones, among which saponins, xanthones and oligosaccharide esters have been identified as the characteristic metabolites of PR.

### 2.1 Saponins

**FIGURE 2:**
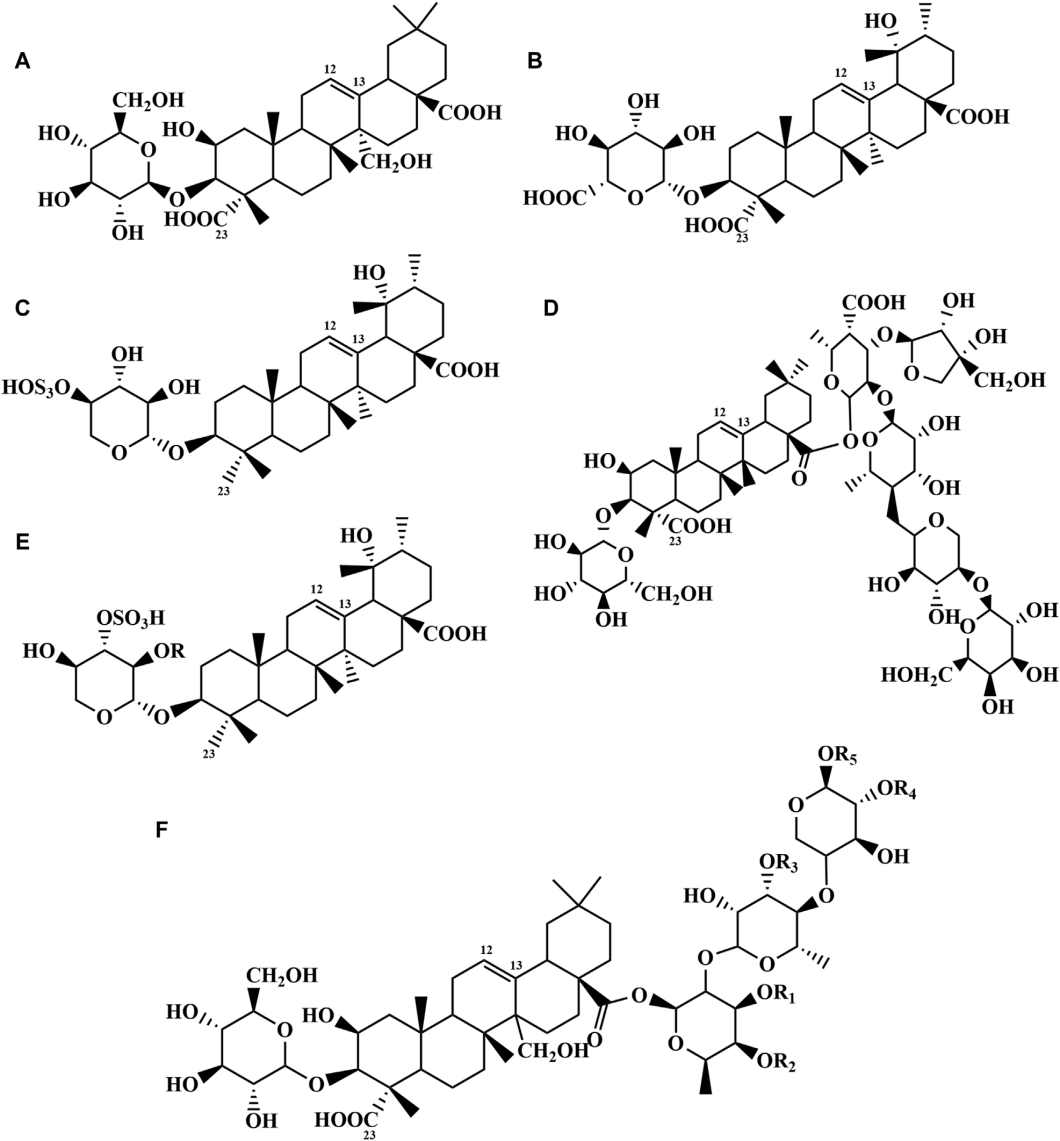
Chemical structures of the saponins in PR (1–50).

Saponins, as one of the main active metabolites of PR, are oleanane type pentacyclic triterpene saponins (Lv et al., 2014). The types of sugars include glucose, rhamnose, xylose, celery sugar, galactose and so on (Han et al., 2010). Triterpenoid saponins are abundant in PR. The total saponins in the leaves of *P. tenuifolia* were 2.46%, and the roots are 3.29% whereas in the *P. sibirica* were 1.50%, and 1.61%, respectively (Jiang et al., 2021). Polygala saponins are different from other polygala aglycones in that the 2-position carbon substituents are different (hydroxyl or ketone), the 12-position and 13-position are unsaturated double bonds, and most of them are double sugar chains. The degree of carbon oxidation at position 23 is different (methyl, aldehyde, hydroxymethyl, carboxyl or methylene), and the number and position of double bonds are different (Fu et al., 2006). At present, more than 50 saponins have been isolated from PR (Table 2; Figure 2). Among them, the Chinese Pharmacopoeia requires that tenuifolin should not be less than 2.0% in the quality control of PR.

**TABLE 2:** Saponins isolated from PR.

NO.	Metabolites	Core	Substituent					Ref.
			R1	R2	R3	R4	R5	
1	Tenuifolin	A	—-	—-	—-	—-	—-	Pelletier and Nakamura (1967)
2	Sibiricasaponin A	B	—-	—-	—-	—-	—-	Ikeya et al. (1991a)
3	Sibiricasaponin C	C	—-	—-	—-	—-	—-	Ikeya et al. (1991a)
4	Sibiricasaponin E	D	—-	—-	—-	—-	—-	Ikeya et al. (1991a)
5	Sibinicasponin B	E	H	—-	—-	—-	—-	Ikeya et al. (1991a)
6	Sibiricasaponin D	E	AC	—-	—-	—-	—-	Ikeya et al. (1991a)
7	Onjisaponin A	F	Rha	MC	Api	H	Gal	Sakuma and Shoji (1982)
8	Onjisaponin B	F	Fuc	MC	H	H	Gal	Sakuma and Shoji (1982)
9	Onjisaponin E	F	H	TC	H	H	Gal	Sakuma and Shoji (1982)
10	Onjisaponin F	F	H	TC	Api	Ara	H	Liu et al. (2007)
11	Onjisaponin G	F	H	TC	Api5HMG	Ara	H	Liu et al. (2007)
12	Onjisaponin J	F	Rha	MC	Api5HMG	Ara	H	Li et al. (2006)
13	Onjisaponin L	F	Rha	MC	Api5HMG	H	Gal	Li et al. (2006)
14	Onjisaponin O	F	Rha	TC	H	H	Gal	Li et al. (2006)
15	Onlisaponin R	F	H	TC	Api	H	Gal	Li et al. (2006)
16	Onjisaponin S	F	Rha	TC	Api	Ara	H	Li et al. (2006)
17	Onjisaponin T	F	Glc6AC	TC	Api	Ara	H	Li et al. (2006)
18	Onjisaponin Fg	F	H	TC	Api5HMG	Ara	H	Li et al. (2006)
19	Onjisaponin Gg	F	H	TC	Api5HMG	H	H	Li et al. (2006)
20	Onjisaponin Ng	F	Rha	MC	Api5HMG	H	H	Li et al. (2006)
21	Onjisaponin Pg	F	Rha	H	Api5HMG	H	Gal	Li et al. (2006)
22	Onjisaponin Qg	F	H	H	Api5HMG	H	Gal	Li et al. (2006)
23	Onjisaponin Sg	F	Rha	TC	Api5HMG	Ara	H	Li et al. (2006)
24	Onjisaponin Tg	F	Glc6AC	TC	Api5HMG	Ara	H	Li et al. (2006)
25	Onjisaponin Ug	F	H	TC	Api5HMG	Ara	H	Li et al. (2006)
26	(E)-Polygalasaponin XXXII	F	Rha	MC	Api	Ara	H	Li et al. (2006)
27	Z)-Onjisaponin J	F	Rha	Z-MC	Api5HMG	Ara	H	Li et al. (2006)
28	(Z)-Onjisaponin L	F	Rha	Z-MC	Api5HMG	H	Gal	Li et al. (2008)
29	Onjisaponin H	F	Rha	MC	Api	H	H	Li et al. (2008)
30	(Z)-Onjisaponin H	F	Rha	Z-MC	Api	H	H	Li et al. (2008)
31	Onjisaponin V	F	TC	H	Api5HMG	H	Gal	Li et al. (2008)
32	Onjisaponin W	F	TC	H	Api5HMG	Ara	H	Li et al. (2011)
33	Onjisaponin X	F	Gal	TC	Api5HMG	Ara	H	Li et al. (2011)
34	Onjisaponin Y	F	Rha	MC	H	H	H	Li et al. (2011)
35	Onjisaponin Z	F	Rha	TC	H	H	H	Li et al. (2011)
36	Onjisaponin Vg	F	H	TC	Api5HMG	H	Gal	Li et al. (2011)
37	Polygalasaponin XXXI	F	H	TC	Api	Ara	H	Li et al. (2011)
38	(E)-Senegzsaponin a	F	H	MC	Api	H	Gal	Li et al. (2011)
39	Onjisaponin Wg	F	TC	H	Api	Ara	H	Li et al. (2011)
40	Onjisaponin MF	F	H	MC	H	H	H	Ling et al. (2013)
41	Onjisaponin TE	F	Rha	H	Api5HMG	H	H	Ling et al. (2013)
42	Onjisaponin TF	F	Rha	H	H	H	H	Feng et al. (2019)
43	Onjisaponin TG	F	H	H	Api5HMG	H	H	Feng et al. (2019)
44	Onjisiponin TH	F	H	TC	Api	H	H	Feng et al. (2019)
45	Polygalhspnin XLV	F	Glc6AC	DC	H	H	Gal	Feng et al. (2019)
46	Polygalasrponin LⅢ	F	Glc6AC	MC	Api	Ara	H	Feng et al. (2019)
47	Myrtifolioside Al	F	Ara	MC	Api	Gal	H	Feng et al. (2019)
48	Desicysenertsapnn	F	H	H	H	H	Gal	Feng et al. (2019)
49	Arillocide D	F	H	H	H	Ara	Gal	Feng et al. (2019)
50	Arillocide A	F	AC	AC	H	H	H	Ikeya et al. (1994)

Rha = a-L-rhamnopyranose; Gal = β-D-galactopyranosyl; Api = β-D-apiofuranosyl; Ara = β-D-arabopyranosyi; Api5HMG, 3-hydroxy-3-methyl-5-penlanoic acid esler-5-β-D-apiofuranosyl; Fuc = β-D-fucopyranosyl; AC, acetyi; DC, 3,4-dimethoxylcinnamoyl; MC = (E)-4-methoxy cinnamoyl: Z-MC = (Z)-4-methoxy cinnamoyl; TC = (E)-3,4,5-trimethoxy cinnamoyl.

### 2.2 Xanthones

**FIGURE 3:**
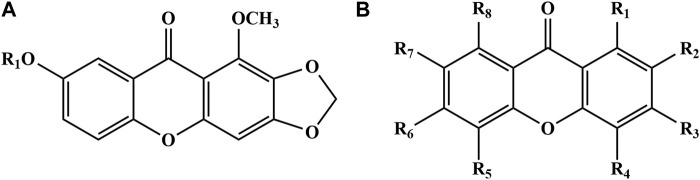
Chemical structures of the xanthones in PR (51–95).

Xanthones, also known as benzochromones, is a kind of yellow or white phenolic metabolites, which has similar color reaction and spectral characteristics with flavonoids (Tan and Lu, 1995). As a kind of active metabolites with high content in PR, the xanthone metabolites are generally divided into simple xanthone, xanthone oxyglycoside, xanthone carboglycoside and BIS xanthone, among which simple ketone metabolites account for the largest proportion (Liu and Ma, 2014). Simple xanthone metabolites refer to a single molecule ketone substituted by a small group. The substituents are mostly hydroxyl, methoxy, and methylenedioxy. The eight positions of the xanthone mother nucleus can be replaced, but the probability of being replaced is different (Yang et al., 2000). The research shows that the substitution probabilities of each position of xanthone are R1 = 97%, R2 = 68%, R3 = 86%, R4 = 20%, R5 = 3%, R6 = 30%, R7 = 70%, R8 = 14% (Zhao et al., 2005). According to the number of oxygen-containing substituents, xanthone metabolites can be divided into five types: di-substituted, tri-substituted, tetra-substituted, penta-substituted and hexa-substituted, and the highest number of oxygen substituents is 6. So far, more than 40 xanthone metabolites have been isolated from PR (Table 3; Figure 3). Among them, Polygalaxanthone III is one of the index metabolites of PR quality control in Chinese Pharmacopoeia.

**TABLE 3:** Xanthones isolated from PR.

NO.	Metabolites	Core	Substituent								Ref.
			R1	R2	R3	R4	R5	R6	R7	R8	
51	1,7-Dimethoxy-2,3- methylenedioxyxanthone	A	OMe	—-	—-	—-	—-	—-			Fujita et al. (1992)
52	7-Hydroxy-1-methoxy-2,3- methylenedioxyxanthone	A	H	—-	—-	—-	—-	—-			Xu et al. (2014)
53	Onjixanthone I	B	OMe	OMe	OMe	H	H	H	OH	H	Sakuma and Shoji (1982)
54	Onjixanthone II	B	OH	OMe	OH	H	H	OH	OMe	H	Sakuma and Shoji (1982)
55	3-Hydroxy-2,8-dimethoxyxanthone	B	H	OMe	OH	H	H	H	H	OMe	Fujita et al. (1992)
56	7-Hydroxy-1,2,3- trimethoxyxanthone	B	OMe	OMe	OMe	H	H	H	OH	H	Fujita et al. (1992)
57	3-Hydroxy-1,2,7- trimethoxyxanthone	B	OMe	OMe	OH	H	H	H	OMe	H	Fujita et al. (1992)
58	6,8-Dihydroxy-1,2,4- trimethoxyxanthone	B	OMe	OMe	H	OMe	H	OH	H	OH	Fujita et al. (1992)
59	6,8-Dihydroxy-1,2,3- trimethoxyxanthone	B	OMe	OMe	OMe	H	H	OH	H	OH	Fujita et al. (1992)
60	Polygalaxanthone III	B	OH	Glc6Api	OH	H	H	OH	OMe	H	Miyase et al. (1999)
61	Sibiricaxanthone A	B	OH	Glc6Api	OH	H	H	H	OH	H	Miyase et al. (1999)
62	Sibiricaxanthone B	B	OH	Glc2Api	OH	H	H	H	OH	H	Miyase et al. (1999)
63	Polygalaxanthone IV	B	OH	H	OMe	H	H	OGlc2Rha	OMe	H	Jiang and Tu (2002a), Jiang et al. (2005)
64	Polygalaxanthone V	B	OH	H	OH	H	H	OGlc2Rha	OMe	H	Jiang and Tu (2002a), Jiang et al. (2005)
65	Polygalaxanthone VI	B	OMe	OMe	OMe	H	H	OGlc	OMe	H	Jiang and Tu (2002a), Jiang et al. (2005)
66	Polygalaxanthone VII	B	OH	OMe	OGlc2Rha	H	H	OH	OMe	H	Jiang and Tu (2002a), Jiang et al. (2005)
67	Polygalaxanthone VIII	B	OH	Glc6Ara	OH	H	H	OH	OMe	H	Jiang and Tu (2002a), Jiang et al. (2005)
68	Polygalaxanthone IX	B	OH	H	OGlc2Rha	H	H	H	OH	H	Jiang and Tu (2002a), Jiang et al. (2005)
69	Polygalaxanthone X	B	OMe	OMe	OMe	H	H	OGlc2Rha	OMe	H	Jiang and Tu (2002a), Jiang et al. (2005)
70	Polygalaxanthone XI	B	OH	Glc2Api	OH	H	H	OH	OMe	H	Jiang and Tu (2002a), Jiang et al. (2005)
71	Lancerin	B	OH	H	OH	Glc	H	H	OH	H	Jiang and Tu (2002a), Jiang et al. (2005)
72	6-Hydroxy-1,2,3,7- tetramethoxyxanthone	B	OMe	OMe	OMe	H	H	OH	OMe	H	Zhou et al. (2014)
73	1,3,7-Trihydroxy-2- methoxyxanthone	B	OH	OMe	OH	H	H	H	OH	H	Zhou et al. (2014)
74	1,2,3,6,7-Pentamethoxyxanthone	B	OMe	OMe	OMe	H	H	OMe	OMe	H	Zhou et al. (2014)
75	1,3,7-Trihydroxy-2,6- dimethanoxyxanthone	B	OH	OMe	OH	H	H	OMe	OH	H	Xu et al. (2014)
76	7-Hydroxy-1-methoxyxanthone	B	OMe	H	H	H	H	H	OH	H	Xu et al. (2014)
77	1,7-Dihydroxy-3,4- dimethoxyxanthone	B	OH	H	OMe	OMe	H	H	OH	H	Xu et al. (2014)
78	Polygalaxanthone lll	B	OH	Glc6Api	OH	H	H	OH	OMe	H	Jiang and Tu (2002b)
79	1,3,6-Trihydroxy-2,7-dimethoxyxanthone	B	OH	OMe	OH	H	H	OH	OMe	H	Ikeya et al. (1991b)
80	1,2,7-Trimethoxy-3-hydroxyxanthone	B	OMe	OMe	OH	H	H	H	OMe	H	Ikeya et al. (1991b)
81	1,2,3,7-Tetramethoxyxanthone	B	OMe	OMe	OMe	H	H	H	OMe	H	Ikeya et al. (1991b)
82	1,7-Dihydroxy-3-methoxyxanthone	B	OH	H	OMe	H	H	H	OH	H	Ikeya et al. (1991b)
83	1,7-Dihydroxy-2,3-dimethoxyxanthone	B	OH	OMe	OMe	H	H	H	OH	H	Fujita et al. (1992)
84	1,7-Dihydroxy-2,3-di-methylene-dioxyxanthone	B	OH	OCH_2_O	H	H	H	OH	H	H	Jiang and Tu (2002b)
85	6-Hydroxy-2,3,6,7-tetramethoxyxanthone	B	H	OMe	OMe	H	H	OMe	OMe	H	Sakuma and Shoji (1982)
86	1,7-Dihydroxyxanthone	B	OH	H	H	H	H	H	OH	H	Hanjiro et al. (1977)
87	1,7-Dimethoxyxanthone	B	OMe	H	H	H	H	H	OMe	H	Hanjiro et al. (1977)
88	1-Hydroxy-3,7-dimethoxyxanthone	B	OH	H	OMe	H	H	H	OMe	H	Hanjiro et al. (1977)
89	1-Hydroxy-3,6,7-trimethoxyxanthone	B	OH	H	OMe	H	H	OMe	OMe	H	Ikeya et al. (1991b)
90	1,3,7-Trihydroxyxanthone	B	OH	H	OH	H	H	H	OH	H	Jiang et al. (2003)
91	1,6,7-Trihydroxy-2,3-dimethoxyxanthone	B	OH	OMe	OMe	H	H	OH	OH	H	Jiang et al. (2003)
92	2,3,8-Trimethoxyxanthone	B	H	OMe	OMe	H	H	H	H	OMe	Fujita et al. (1992)
93	1,3,6,7-Tetramethoxyxanthone	B	OMe	H	OMe	H	H	OMe	OMe	H	Fujita et al. (1992)
94	1,3,7-Trimethoxyxanthone	B	OMe	H	OMe	H	H	H	OMe	H	Jiang et al. (2003)
95	7-O-methylmangiferin	B	OH	Glc	OH	H	H	OH	OMe	H	Jiang et al. (2003)

### 2.3 Oligosaccharide esters

**FIGURE 4:**
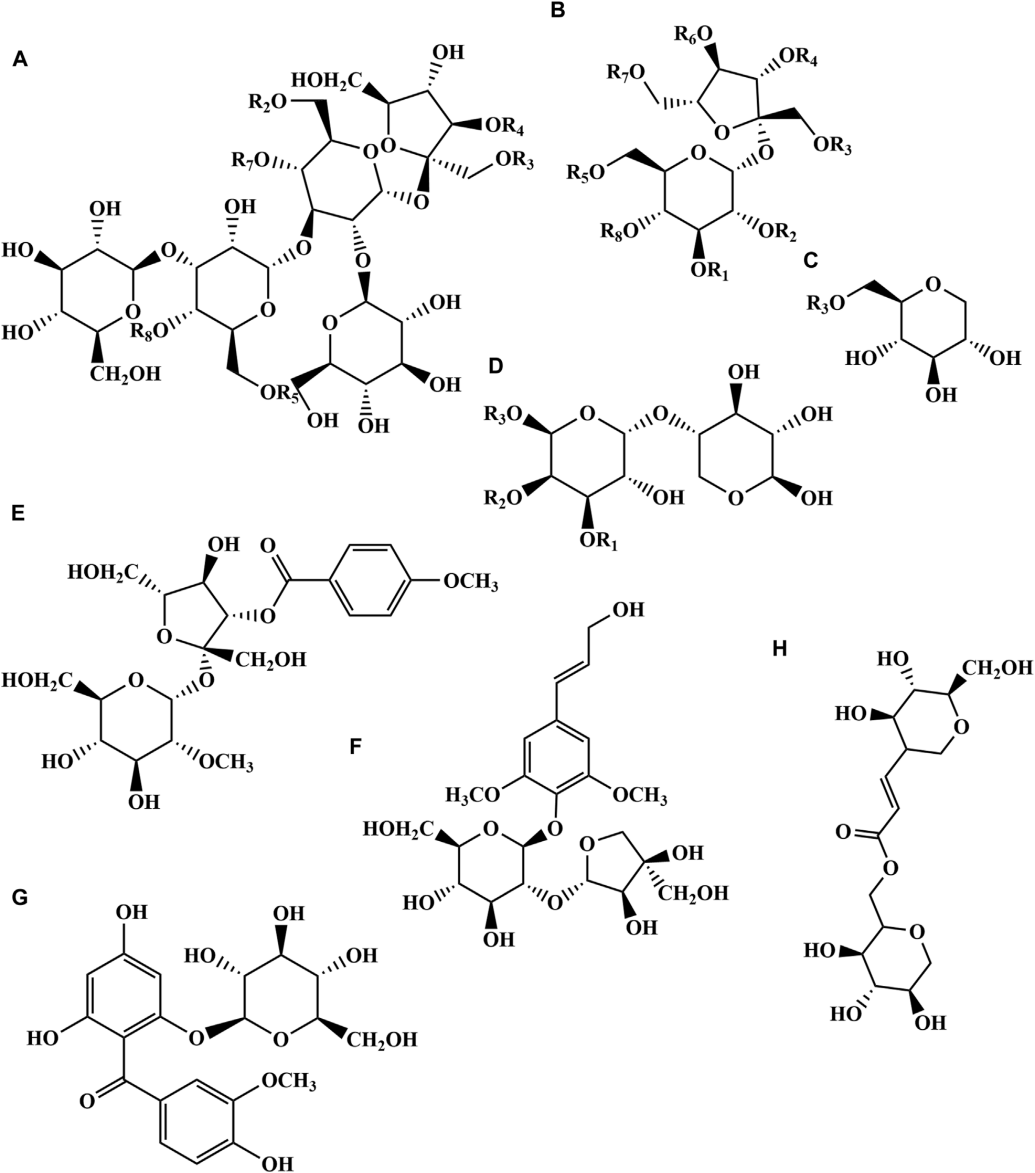
Chemical structures of the oligosaccharide esters in PR (96–131).

Oligosaccharide ester is a unique metabolite found in PR, which is mainly concentrated in the root (Miyase et al., 1999). Oligosaccharide esters mainly use sucrose as the mother nucleus, connect glucose or rhamnose with various forms of glycosidic bonds, and then form esters with organic acids such as acetic acid, benzoic acid and phenylacrylic acid and their derivatives (Miyase et al., 1991; Zhao et al., 2020). With the in-depth study of the metabolites and pharmacological effects of PR, oligosaccharide metabolites have become an important part that cannot be ignored. At present, more than 30 oligosaccharide esters have been isolated and identified from PR (Table 4; Figure 4). Oligosaccharide esters are widely found in plants, but sugar esters above trisaccharides are only found in Polygalaceae, which are considered to be unique metabolites of Polygalaceae. In Chinese Pharmacopoeia, 3,6′-disinapoyl sucrose is one of the index metabolites of PR quality control.

**TABLE 4:** Oligosaccharide esters isolated from PR.

NO.	Metabolites	Core	Substituent								Ref.
			R1	R2	R3	R4	R5	R6	R7	R8	
96	Tenuifoliose A	A	—-	a	e	b	a	—-	i	a	Miyase and Ueno (1993)
97	Tenuifoliose B	A	—-	H	e	b	a	—-	i	a	Miyase and Ueno (1993)
98	Tenuifoliose C	A	—-	H	e	b	a	—-	i	H	Miyase and Ueno (1993)
99	Tenuifoliose D	A	—-	a	e	b	a	—-	i	H	Miyase and Ueno (1993)
100	Tenuifoliose E	A	—-	a	e	b	H	—-	i	H	Miyase and Ueno (1993)
101	Tenuifoliose F	A	—-	a	e	b	a	—-	m	a	Miyase et al. (1991)
102	Tenuifoliose G	A	—-	a	e	b	a	—-	m	H	Miyase et al. (1992)
103	Tenuifoliose H	A	—-	a	e	b	a	—-	e	a	Miyase et al. (1992)
104	Tenuifoliose I	A	—-	a	e	b	a	—-	e	H	Miyase et al. (1992)
105	Tenuifoliose J	A	—-	H	e	b	a	—-	e	a	Miyase et al. (1992)
106	Tenuifoliose K	A	—-	H	e	b	a	—-	e	H	Miyase et al. (1992)
107	Tenuifoliose L	A	—-	a	e	b	a	—-	n	a	Miyase et al. (1992)
108	Tenuifoliose M	A	—-	a	e	b	a	—-	n	H	Miyase et al. (1992)
109	Tenuifoliose N	A	—-	a	i	b	a	—-	i	a	Miyase et al. (1992)
110	Tenuifoliose O	A	—-	H	i	b	a	—-	i	a	Miyase et al. (1992)
111	Tenuifoliose P	A	—-	H	i	b	a	—-	i	H	Miyase et al. (1992)
112	Tenuifoliose Q	A	—-	H	e	b	a	—-	n	a	Miyase et al. (1991)
113	Tenuifoliside A	B	H	H	H	l	g	H	H	H	Yang et al. (2002)
114	Tenuifoliside B	B	H	H	H	j	g	H	H	H	Yang et al. (2002)
115	Tenuifoliside C	B	H	H	H	j	j	H	H	H	Yang et al. (2002)
116	Tenuifoliside D	C	—-	—-	l	—-	—-	—-	—-	—-	Yang et al. (2002)
117	Tenuifoliside E	B	H	a	H	h	a	H	H	a	Ikeya et al. (1991b)
118	3,6′-Disinapoyl sucrose	B	H	H	H	j	j	H	H	H	Ikeya et al. (1991b)
119	Sibiricose A1	B	H	H	H	H	a	H	H	a	Jiang and Tu (2003)
120	Sibiricose A2	B	H	H	H	H	j	H	H	H	Jiang and Tu (2003)
121	Sibiricose A3	B	H	H	H	g	l	H	H	H	Jiang and Tu (2003)
122	Sibiricose A4	B	H	H	H	j	H	H	H	j	Jiang and Tu (2003)
123	Sibiricose A5	B	H	H	H	i	H	H	H	H	Jiang and Tu (2003)
124	Sibiricose A6	B	H	H	H	j	H	H	H	H	Jiang and Tu (2003)
125	Polygalatenoside A	D	H	H	b	—-	—-	—-	—-	—-	Miyase et al. (1999)
126	Polygalatenoside B	D	b	H	H	—-	—-	—-	—-	—-	Miyase et al. (1999)
127	Polygalatenoside C	D	H	b	H	—-	—-	—-	—-	—-	Miyase et al. (1999)
128	Polygalatenoside D	E	—-	—-	—-	—-	—-	—-	—-	—-	Miyase et al. (1999)
129	Polygalatenoside E	F	—-	—-	—-	—-	—-	—-	—-	—-	Miyase et al. (1999)
130	Sibiriphenone A	G	—-	—-	—-	—-	—-	—-	—-	—-	Zhou et al. (2014)
131	Sibiricose A7	H	—-	—-	—-	—-	—-	—-	—-	—-	Zhou et al. (2014)

a = acetyl; b = benzoyl; e = (E)-p-coumaroyl; g = p-hydroxylbenzoyl; j = (E)-sinapoy; i = (E)-feruloyl; l = (E)-3,4,5-trimethoxycinnamoyl; m = 4-O-a-L-rhamnopyranosyl-(E)-feruloyl; n = 4-O-a-Lrhamnopyranosy-(E)-p-coumaroyl.

### 2.4 Other metabolites

**FIGURE 5:**
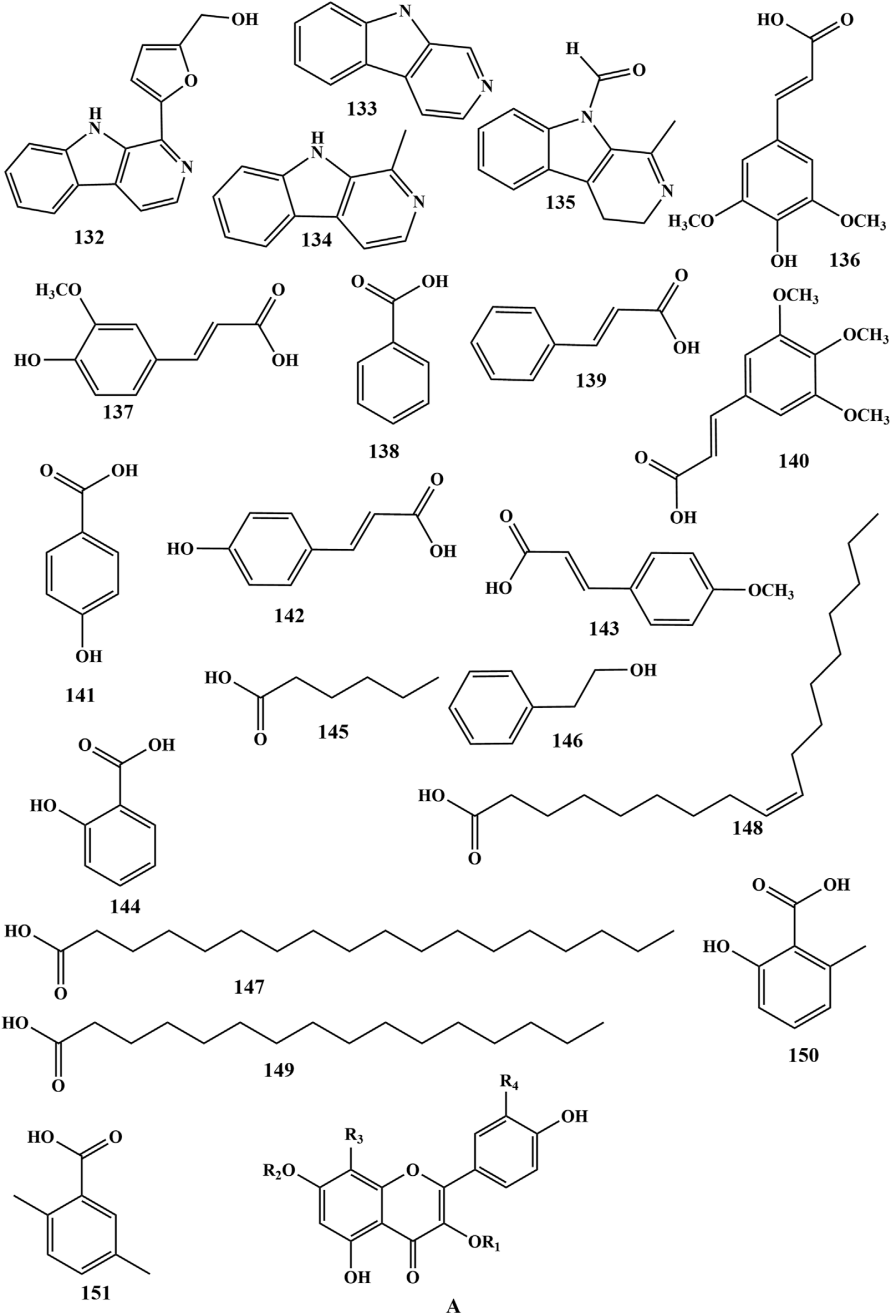
Chemical structures of other metabolites in PR (132–161).

Alkaloids are widely found in plants and have significant and unique biological activities. At present, seven alkaloids have been isolated from the roots of *P. tenuifolia*, including harman, noharman, N9-formylharman, 1-butoxycarbonyl-β-carboline, one-ethoxycarbonyl-β-carboline, 1-methoxycarbonyl-β-carboline and perlolyrine (Table 5; Figure 5). In addition, organic acids and volatile oils are also important metabolites of PR. 3,4,5-trimethoxycinnamic acid and ferulic acid were isolated from the roots of *P. tenuifolia*. Ursolic acid, 2α, 3β, 19α-trihydroxyurs-12-en-23,28-dicarboxylic acid, 3β, 19α-dihydroxyurs-12-en-23,28-dicarboxylic acid, 3β, 19α-dihydroxyolean-12-en-23,38-dicarboxylic acid and other metabolites were also isolated and identified from the aerial parts of *P. sibirica*. In addition to the above metabolites, PR also identified flavonoids, coumarins, phenylpropanoids, steroids, and inorganic metal elements such as Zn, K, Ca, Cu, Fe, Mn, and Mg (Table 5; Figure 5).

**TABLE 5:** Other metabolites isolated from PR.

NO.	Metabolites	Core	Substituent				Ref.
			R1	R2	R3	R4	
132	Perlolyrine	—-	—	—	—	—	Jin and Piao (1993)
133	Norharman	—	—	—	—	—	Jin and Piao (1993)
134	Harman	—	—	—	—	—	Jin and Piao (1993)
135	N9-formylharman	—	—	—	—	—	Jin and Piao (1993)
136	Sinapic acid	—-	—-	—-	—-	—-	Song et al. (2016)
137	Ferulic acid	—-	—-	—-	—-	—-	Song et al. (2016)
138	Benzoic acid	—-	—-	—-	—-	—-	Song et al. (2016)
139	Cinnamic acid	—-	—-	—-	—-	—-	Song et al. (2016)
140	3,4,5-Trimethoxycinnamic acid	—-	—-	—-	—-	—-	Song et al. (2016)
141	p-Hydroxybenzoic acid	—-	—-	—-	—-	—-	Song et al. (2016)
142	p-Coumaric acid	—-	—-	—	—	—-	Song et al. (2016)
143	p-Methoxy cinnamic acid	—-	—-	—-	—-	—-	Song et al. (2016)
144	O-Hydroxybenzoic acid	—-	—-	—-	—-	—-	Jiang et al. (2011)
145	Hexanoic acid	—-	—-	—-	—-	—-	Wu (2010)
146	Phenethyl alcohol	—-	—-	—-	—-	—-	Wu (2010)
147	Stearic acid	—-	—-	—-	—-	—-	Wu (2010)
148	Oleic acid	—-	—-	—-	—-	—-	Wu (2010)
149	Palmitic acid	—-	—-	—-	—-	—-	Wu (2010)
150	Methylsalicylic acid	—-	—-	—-	—-	—-	Wu (2010)
151	2,5-Dimethylbenzaldehyde	—-	—-	—-	—-	—-	Wu (2010)
152	Linarin	A	H	Xy2Rha	H	OH	Shi et al. (2013)
153	Isorhamnetin	A	H	H	H	OMe	Shi et al. (2013)
154	Isorhamnetin-3-O-β-D-glucopyranoside	A	Glc	H	H	OMe	Shi et al. (2013)
155	Isorhamnetin-3-O-β-D- galactopyranoside	A	Gal	H	H	OMe	Shi et al. (2013)
156	Quercetin-3-O-β-D-glucopyranosyl (1→2)-β-D-galactopyranoside	A	Glc2Gal	H	H	OH	Shi et al. (2013)
157	Quercetin-3-O-β-D-glucopyranosyl (1→2)-β-D-glucopyranoside	A	Glc2Gal	H	H	OH	Shi et al. (2013)
158	Quercetin-3-O-β-D-glucopyranoside	A	Gal	H	H	OH	Shi et al. (2013)
159	5,7-Dihydroxy-8-methxoyflavone-7-O- β-D-glucuronoside	A	H	GlcA	OMe	OH	Shi et al. (2013)
160	Kaempferol	A	H	H	H	H	Shi et al. (2013)
161	Quercetin	A	H	H	H	OH	Shi et al. (2013)

The metabolites of PR are complex, and more than 160 metabolites have been isolated and identified. However, at present, the research on the metabolites of PR at home and abroad mainly focuses on saponins, oligosaccharide esters and crude extracts, and most other metabolites have not been further studied. In this paper, the metabolites of PR are reviewed, which is helpful to further study the material basis of PR and provide scientific basis for the development and clinical application of new drugs.

## 3 Pharmacology

Modern pharmacological studies have shown that PR has a wide range of pharmacological activities, including the protection of the nervous system, immune system, cardiovascular system and respiratory system. Especially for the central nervous system. It is often compatible with *Acorus tatarinowii* Schott, *Panax ginseng* C. A. Mey., *Poria cocos* (Schw.) Wolf and other drugs.

### 3.1 Effects on the nervous system

The research of modern pharmacology on PR has been continuing. Through extensive research, it has been found that the effects of *P. tenuifolia* mainly focus on the central nervous system, especially sedative hypnosis, neuroprotection, improving cognitive impairment and improving learning and memory ability (Table 6).

**TABLE 6:** Effects of RP on the nervous system.

Pharmacological effects	Extracts/metabolites	Model	Dose range tested	Mechanism	Ref.
Sedative hypnotic effect	BuOH sol. Fract	Mice	6.25 mg/kg	Inhibition of cAMP phosphodiesterase	Nikaido et al. (1982)
	Onjisaponins E	Mice	20 mg/kg	Inhibition of cAMP phosphodiesterase	Nikaido et al. (1982)
	Onjisaponins F	Mice	20 mg/kg	Inhibition of cAMP phosphodiesterase	Nikaido et al. (1982)
	Onjisaponins G	Mice	20 mg/kg	Inhibition of cAMP phosphodiesterase	Nikaido et al. (1982)
	3,4,5-Trimethoxycinnamic Acid	ST rats	50 μg/rat	Inhibit the synthesis and secretion of norepinephrine in locus coeruleus neurons	Kawashima et al. (2004)
	Polygalasaponins	TO mice	25, 50, 100 mg/kg	Antagonism of dopamine and 5-HT receptors	Chung et al. (2002)
	Tenuifolin	ICR mice	20, 40, 80 mg/kg	Activation of GABAergic system, inhibition of noradrenergic system	Cao et al. (2016)
Learning and Memory improvements	Tenuigenin	ICR mice, KM mice	4 mg/kg	Inhibition of AChE activity, antioxidant, enhance synaptic plasticity	Huang et al. (2013)
	Tenuigenin	SAM mice	500 mg/kg	Regulate monoamine neurotransmitter content	Zheng et al. (2010)
	BT-11	Rats	10 mg/kg	Improve glucose utilization and increase the total NCAM level	Shin et al. (2009a)
	Polygalasaponin XXXII	C57BL/6J mice, Wistar rats	0.125, 0.5, 2 mg/kg	Improvement of synaptic transmission, activation of the MAP kinase cascade and enhancement of the level of BDNF	Xue et al. (2009)
Neuroprotective effects	OnjisaponinsV-Z	PC12 cell	10^–5^ mol/L	—-	Li et al. (2008)
	Tenuifolin	COS-7 cells	0.5, 1.0, 2.0 μg/mL	Inhibition of β-site APP lyase	Lv et al. (2009)
	Tenuigenin	SD rats	18.5, 37.0, 74.0 mg/kg	Downregulate protein kinase A, upregulation of PP-2A protein and inhibition of excessive phosphorylation of tau protein Ser396 site	Xu et al. (2012)
	Senegenin	SD rats	300 mg/kg	Increased expression of NR2B	Yuan et al. (2012)
	Tenuigenin	C57BL/6J mice	25, 50 mg/kg	Inhibition of NLRP3 inflammasome activation	Fan et al. (2017)
Antidepressant effects	3,6′-Disinapoyl sucrose	SD rats	10, 20 mg/kg	Increased expressions of noradrenergic-regulated plasticity genes and neurotrophic factor	Hu et al. (2010)
	Extract of PR	ICR mice and Wistar rats	0.13–1.0 g/kg and 0.5, 1 g/kg	Promoting autophagy and inhibiting neuroinflammation	Zhou et al. (2021)
	Extract of PR	C57BL/6 mice	1, 10, 100 mg/kg	Increase the expression of BDNF and BAG1	Han et al. (2021)
	TEA	PC12 cells	0.53, 13.2, 132 μM	Promotes Ca^2+^release	Xu et al. (2008)

#### 3.1.1 Sedative hypnotic effect

PR is a commonly used traditional Chinese medicine for the treatment of insomnia. Early studies have confirmed that the chloroform layer and n-butanol layer from the water or methanol extract of *P. tenuifolia* root showed strong inhibitory activity against cyclic adenosine monophosphate (cAMP) phosphodiesterase, and the *n*-butanol part of those showed stronger inhibitory activity against cAMP phosphodiesterase. When the dose was 100 μg/mL, the inhibition rate was 73.3%. The active metabolites oleic acid and tenuigenin were obtained from these two parts. The IC_50_ values of Onjisaponins E, F and G were close to that of the papaverine which was the positive drug, while the activity of Onjisaponin A was higher than that of papaverine (Nikaido et al., 1982). Although this study confirms the therapeutic effect of PR on insomnia, there is a lack of research on its mechanism of action.

Saponins are the main bioactive metabolites in PR, which have the potential to be developed as sedative hypnotics. Previous studies indicated that polygalasaponins (25–500 mg/kg) could produce dose-related reductions in climbing behavior, 5-hydroxytryptamine syndrome and hyperactivity disorder induced by apomorphine, 5-hydroxytryptamine (5-HTP) and MK-801(Chung et al., 2002). Tenuifolin could significantly prolong total sleep time by increasing the amount of non-rapid eye movements (NREM) and rapid eye movement (REM) sleep. The hypnotic effects of tenuifolin were assessed by electroencephalogram (EEG) and electromyogram (EMG) analysis. Double-staining immunohistochemistry test was performed to evaluate the neuronal activity of sleep-wake regulating brain areas. High performance liquid chromatograph-electrochemical detection (HPLC-ECD) and ultrafast liquid chromatography-mass spectrometry (UFLC-MS) were used for the detection of neurotransmitters. Locomotor activity was measured by Open-field Test. Experimental results found that tenuifolin could increase the positive rate of c-Fos in GABAergic NREM sleep-promoting neurons in the ventrolateral preoptic area (VLPO), and cholinergic REM sleep-promoting neurons in the laterodorsal tegmental area (LDT) and the pontomesencephalic tegmental area (PPT), and reduce the positive rate of c-Fos in wake-promoting neurons. Tenuifolin also significantly reduced noradrenaline (NA) levels in the locus coeruleus (LC), VLPO, PPT, and LDT, increased GABA levels in VLPO, LC, and perifornical area (Pef), and increased acetylcholine (Ach) levels in LDT and PPT, and had no effect on voluntary movement. The above results indicate that tenuifolin can significantly enhance sleep in mice, revealing that PR has the potential to be developed as a sedative hypnotic drug (Cao et al., 2016). However, these studies lack comparative studies of positive drugs and clinical validation. Future scholars should further analyze the effects of saponins on other hypnotic and sedative targets to improve the study of its mechanism.

In 2004, an organic acid isolated from PR, 3,4,5-trimethoxycinnamic acid (TMCA), could significantly inhibit the synthesis and secretion of norepinephrine (NE) in locus coeruleus neurons, and then had a sedative effect on the stress rat model induced by intracerebroventricular injection of corticotropin-releasing hormone (CRH) (Kawashima et al., 2004). However, this study lacks the support of positive control experiments.

#### 3.1.2 Learning and memory improvements

Treasury of Words on the Materia Medica takes PR as a specific drug for the treatment of amnesia, and clearly points out that “the power of PR is dedicated to benefiting the essence and strengthening the will, so it has a special effect for the treatment of amnesia”. Modern pharmacological research also shows that PR has pharmacological effects such as anti-dementia and improving learning and memory ability. The ability of PR water extract to affect the memory of mice was studied by *in vivo* animal experiments. The learning and memory ability of mice was observed by Morris water maze, and the superoxide dismutase (SOD) activity and malondialdehyde (MDA) content in brain tissue were determined. The results indicated that after 21 days of intragastric administration of PR’s water extract, the latency to find the platform of mice was significantly shortened, the number of crossing the platform was significantly increased, the activity of superoxide dismutase (SOD) in brain tissue was significantly increased, and the content of malondialdehyde (MDA) was significantly reduced, suggesting that PR’s water extract could enhance the learning and memory ability of mice (Wang et al., 2012). However, this study did not systematically screen natural small molecule metabolites in PR that can improve learning and memory ability.

The effects of saponins in PR on learning and memory have been studied extensively. Tenuigenin (TEN) was found to exert memory-enhancing effects by reducing the activity of acetylcholinesterase (AChE) and the concentration of MDA, increasing the activity of SOD in hippocampus, increasing the amplitude of synaptic transmission and field excitatory postsynaptic potential (fEPSP) (Huang et al., 2013). In another study, the ability of TEN to improve learning and memory was evaluated using SAM series aging mice. The results revealed that TEN could significantly increase the contents of neurotransmitters 5-HT, 5-hydroxyindoleacetic acid (5-HIAA), dopamine (DA) and NE in the brain of rats, and improve the learning and memory ability of brain aging rats (Zheng et al., 2010). However, the lack of positive controls is the limitation of this study. In addition, the biochemical indicators of this study are single, and there is no in-depth study on the mechanism of TEN to improve learning and memory ability. Polygalasaponin XXXII could improve the learning and memory ability of the hippocampus by improving synaptic transmission, activating mitogen-activated protein kinase (MAPK) cascade and increasing brain-derived neurotrophic factor (BDNF) levels (Xue et al., 2009).

Another PR metabolite, an extract of *P. tenuifolia* (BT-11) could repair stress-induced memory defects by increasing the utilization of glucose in the brain of mice and the level of nerve cell adhesion factor (Shin et al., 2009a). BT-11 could not only improve the cognitive ability of the elderly (Shin et al., 2009b), but also improve the memory of adults (Lee et al., 2009). Although the effects of PR and its metabolites on improving learning and memory ability have been studied, its mechanism and effective dose need to be further studied.

#### 3.1.3 Neuroprotective effect

Due to the aging of the population and various social factors, the incidence of neurological diseases has risen sharply, such as cerebrovascular diseases, Alzheimer‘s disease (AD) and Parkinson‘s disease (PD), which seriously affect the quality of patients’ life and bring a huge burden to the family and society.

The antagonistic effect of Onjisaponins V-Z on the toxicity of pheochromocytoma cells (PC12) induced by glutamate and serum deficiency was studied. The results showed that when the serum concentration decreased or excessive glutamate was added to the medium, the survival rate of PC12 cells decreased significantly, while TEN increased the survival rate (Li et al., 2008). In 2009, another study on the neuroprotective effect of saponins showed that tenuifolin may play a role by inhibiting the β-site APP lyase, thereby reducing the Aβ secretion of COS-7 cells (Lv et al., 2009). However, these two studies on the neuroprotective effect of PR lack positive control and dose-dependent analysis.

It is necessary to study the intervention effect of TEN on AD rats, because TEN could downregulate the expression of protein kinase A, upregulate the expression of PP-2A protein, inhibit the hyperphosphorylation of tau protein Ser396 site in brain neurons of AD rats, and reduce the damage to nerve cells (Xu et al., 2012). In addition, TEN has also been revealed to nourish nerve cells by regulating MAPK/NF-κB, Nrf2/HO-1, PI3K/Akt, ROS/Ca^2+^ and other pathways, inhibit inflammation and exert neuroprotective effects. In addition, behavioral analysis, high performance liquid chromatography, immunohistochemistry and enzyme-linked immunosorbent assay were used to observe the effect of TEN on lipopolysaccharide (LPS) -induced PD model. It was reported that TEN could inhibit the activation of NLRP3 inflammasome, the division of caspase-1 and the secretion of IL-1β (Yuan et al., 2012), significantly improve the degeneration of dopaminergic neurons for the treatment of PD. (Fan et al., 2017). The pathogenesis of PD and AD is complex and the pathways are diverse. Therefore, more experiments are needed to study the role and mechanism of TEN in neurodegenerative diseases.

#### 3.1.4 Antidepressant effects

The pathogenesis of depression is complex, and there is no consistent theoretical concept in clinical practice. Therefore, it is urgent to seek effective drugs for the treatment of depression in clinical practice. In recent years, more and more attention has been paid to the antidepressant effect of PR. To study the antidepressant effect of PR extracts in behavioral despair mice and chronic restraint stress (CRS)-induced rats. The results showed that RP could reduce the immobility time of mice in forced swimming test (FST) and reverse the abnormal behavior of CRS-induced rats in sucrose preference test (SPT), novelty-suppressed feeding test (NSFT), open field test (OFT) and FST. Also, RP could enhance the expression of LC3-II and Beclin1 in mouse cortex and rat prefrontal cortex (PFC), reduce the level of p62, and regulate the dysfunction of AMPK-mTOR pathway in PFC of CRS rats, thus exhibiting antidepressant effects (Zhou et al., 2021). Another study found that RP significantly improved the working memory, situational memory and despair-related behaviors of estrogen-depleted mice, increased the expression of BDNF in the prefrontal cortex and the expression of BAG1 in the hippocampus, indicating that RP could improve the cognitive and depressive symptoms of postmenopausal women (Han et al., 2021). However, these two studies did not systematically screen out the antidepressant metabolites in PR.

In 2008, the antidepressant activity of sibiricose A5 and tenuifoliside A in glutamate-treated PC12 cells was studied. The antidepressant effect of sibiricose A5 and tenuifoliside A was achieved by reversing the Ca^2+^ overload induced by glutamate in PC12 cells and promoting the release of Ca^2+^ in PC12 cells, thereby increasing the release of monoamine neurotransmitters (Xu et al., 2008). 3,6′-disinapoyl sucrose in *P. tenuifolia* could inhibit the increase of plasma cortisol level by increasing the expression of four neural plasticity genes in rats or by increasing SOD activity, thus showing antidepressant effect (Hu et al., 2010). However, in these two studies, the research on the antidepressant mechanism of PR metabolites is relatively shallow, and further research is needed to determine the antidepressant activity of PR metabolites.

### 3.2 Effect on the immune system

As a commonly used traditional Chinese medicine, PR has many pharmacological effects such as immune regulation, anti-inflammation and anti-oxidation. It has a regulatory effect on the immune system, can enhance the immune function of the body and improve the disease resistance of the body. It can be used clinically to treat various immune-related diseases, such as autoimmune diseases and tumors. In particular, TEN has immune adjuvant activity and can enhance immunity (Table 7).

**TABLE 7:** Effects of RP on the immune system.

Pharmacological effects	Extracts/metabolites	Model	Dose range tested	Mechanism	Ref.
Anti-inflammatory effect	Tenuigenin	BV2 cells	2 μM	Inhibition of COX-2 mRNA and COX-2 protein	Pi et al. (2020)
	Tenuigenin	BV2 cells	1, 2, 4 μM	Activation of NRF2-mediated Ho-1 signaling pathway	Wang et al. (2017a)
	Tenuigenin	RAW 264.7 cells	1.86, 3.72 μM	Inhibition of iNOS and COX-2 expression via downregulation of the MAPK and NF-κB, and upregulation of the Nrf2/HO-1 signaling pathways	Lv et al. (2016)
	Tenuigenin	Osteoarthritis chondrocyte	2, 4, 8 μg/mL	Inhibition of PI3K/AKT/NF-κB signal transduction pathway	Wang et al. (2016)
	Tenuigenin	BALB/c mice	2, 4, 8 mg/kg	Inhibition of TLR4/NF-κB signaling pathway	Fu et al. (2016)
Antiviral effect	Tenuigenin	HepG2 cells	0.01–1 μg/mL	Inhibition of IL-1α	Koo et al. (2000)
	3,4,5-Trimethoxycinnamic acid	CIK cells, GCRV	1, 10, 100 mg/kg	Increase the expression of Mx1, IL-1β, TNFα and MyD88	Yu et al. (2014)
	1,5-Anhydro-D-glucitol	CIK cells, GCRV	1, 10, 100 mg/kg	Increase the expression of Mx1, IL-1β, TNFα and MyD88	Yu et al. (2014)
	Polygalasaponin F	BALB/C mice	50, 100, 200 mg/kg	Inhibition of Raf/MEK/ERK and NF-κB expression	(Ye et al., 2020)
Antitumor effect	PTP	SKOV3 cells	0, 20, 40 μg/mL	Depletes glutathione (GSH) and intracellular reactive oxygen species (ROS)	Xin et al. (2012a)
	PTP	BALB/c nude mice	10, 20, 40 mg/kg	Depletes glutathione (GSH) and intracellular reactive oxygen species (ROS)	Xin et al. (2012a)
	PTPa, PTPb	BALB/c nude mice	50, 100 mg/kg	—-	Xin et al. (2012b)
	PTPa, PTPb	A549 cells	0–200 mg/mL	—-	Xin et al. (2012b)

#### 3.2.1 Anti-inflammatory effects

PR has anti-inflammatory effect, which can reduce the inflammatory response in autoimmune diseases and alleviate the disease. Neuroinflammatory reactions occur in the brain of AD patients, and Aβ, as a pro-inflammatory factor, indirectly activates some inflammatory factors. At present, activated microglia, astrocytes and a variety of immune response products, such as IL-1β, IL-6, TNF-α, have been detected in the brain of AD patients. It was found that TEN exerted anti-inflammatory effect by inhibiting COX-2 mRNA and COX-2 protein and activating NRF2-mediated Ho-1 signaling pathway (Pi et al., 2020). However, this study lacked validation at the animal level, which limited the development and application of PR in the treatment of inflammatory diseases.

At present, there are many studies on the anti-inflammatory mechanism of TEN. TEN showed anti-inflammatory effects by down-regulating the production of prostaglandin E2 (PGE2) and NO, inhibiting the expression of iNOS and COX-2, inhibiting the phosphorylation of JNK1/2, ERK1/2, p38 and NF-κB (p65), blocking the phosphorylation and degradation of IκBα, and up-regulating the Nrf2/HO-1 signaling pathway (Lv et al., 2016). Although the anti-inflammatory mechanism was studied in detail in this study, the main limitation was the lack of positive controls. TEN could inhibit IL-1β induced inflammation in human osteoarthritis chondrocytes by inhibiting the PI3K/AKT/NF-κB signaling pathway (Wang et al., 2016). TEN could also prevent LPS-induced AKI by inhibiting the TLR4/NF-κB signaling pathway (Fu et al., 2016).

#### 3.2.2 Antiviral effect

PR has a significant antiviral effect and can be used to treat viral diseases such as influenza and hepatitis. Compared with the brain protection effect of PR, there are few studies on its antiviral effect. The aqueous extract from PR could prevent and treat hepatitis C virus infection. It has been confirmed that the aqueous extract from PR (0.01–1 μg/mL) dose-dependently inhibited ethanol-induced IL-1α secretion, inhibited HepG2 cell apoptosis and thus inhibited ethanol-induced cytotoxicity (Koo et al., 2000). Subsequently the antiviral ability of 3,4,5-trimethoxycinnamic acid and 1,5-anhydro-D-glucitol isolated from PR was evaluated *in vitro* and *in vivo*. *In vitro* experiments showed that the two metabolites upregulated Mx1, IL-1β, TNFα and MyD88 in different degrees in C. idella kidney cell, and had antiviral activity *in vitro*. In the *in vivo* insecticidal test, 3,4,5-trimethoxycinnamic acid showed higher antiviral activity than 1,5-anhydro-D-glucitol (Yu et al., 2014). However, this study lacks the support of positive control and clinical experimental data.

In 2020, mice were infected intranasally with fifteen 50% mouse lethal challenge doses (MLD50) of influenza virus. BALB/c mice were treated with PSF or oseltamivir (oral administration) 2 h after infection, and the corresponding treatment was given 5 d after infection. 6 days after infection, relevant samples were collected, body weight and lung wet weight were measured, and viral load, cytokines, prostaglandins, pathological changes and cell pathway protein expression in lung tissue were detected. The results show that polygalasaponin F could also enhance the protective effect of IAV infection in mice by inhibiting the expression of Raf/MEK/ERK and NF-κB (Ye et al., 2020).

#### 3.2.3 Antitumor effect

PR plays an important role in tumor immunotherapy. PR can improve the recognition and killing ability of tumor cells by enhancing the immune function of the body. In addition, some metabolites of PR can inhibit the growth and proliferation of tumor cells, which is conducive to the treatment of tumors.

Its effect on the growth of human ovarian cancer cells SKOV3 was studied *in vitro* and in ovarian cancer rats. Studies have found that PTP, a polysaccharide isolated from PR, could cause apoptosis by depleting glutathione (GSH) and intracellular reactive oxygen species (ROS) in cancer cells, thereby inhibiting the proliferation of SKOV3 cells (Xin et al., 2012a). PTP could not only inhibit ovarian cancer but also could be used to prevent the occurrence of lung cancer (Xin et al., 2012a). This study is only carried out at the cellular level *in vitro*, and the mechanism of action and protein targets *in vivo* still need to be further explored. The antitumor activity of two acidic polysaccharides PTPa and PTPb was evaluated *in vitro* and *in vivo*. It was found that PTPa and PTPb could significantly inhibit the growth of A549 cells *in vitro*. The treatment of tumor-bearing mice with two acidic polysaccharides could lead to the increase of SOD and catalase (CAT) activity and the decrease of thiobarbituric acid reactive substances (TBARS) level, showed obvious anti-tumor activity *in vivo* (Xin et al., 2012b).

### 3.3 Effects on cardiovascular system

Cardiovascular diseases have become the first cause of death and disability in the world. Bad habits in daily life, such as smoking, unhealthy diet, obesity, lack of exercise and excessive drinking, may lead to cardiovascular disease. In recent years, many experts and scholars at home and abroad are working to find effective methods to prevent and treat cardiovascular diseases. Therefore, it is one of the hot topics for scientists to find natural drugs with high efficiency and low toxicity to prevent and treat cardiovascular diseases. The pharmacological effects of PR on the cardiovascular system are shown in Table 8.

**TABLE 8:** Effects of RP on the cardiovascular system.

Pharmacological effects	Extracts/metabolites	Model	Dose range tested	Mechanism	Ref.
Hypotensive effect	Presenegenin	SD rats	19, 76 µM	—-	Peng (1999)
Lipid-regulating effect	Extract of PR	3T3-L1 cell	500 μg/mL	Inducing the expression of the master transcription factor PPARα	Wang et al. (2017b)
Cardiovascular effect	3,4,5-Tri methoxy cinnamate	Single ventricular myocytes	15, 30 µM	Inhibition of calcium channel	Zhao et al. (2013)
	Tenuigenin	SD rats	26 mg/kg	The formation of antioxidant free radicals and NO free radicals	Guo and Shen (2005)

#### 3.3.1 Antihypertensive effect

PR could reduce the mean arterial pressure recorded in the left common carotid artery of anesthetized rats and the blood pressure of awake rats and renal hypertensive rats (RVHR). The study of the mechanism of action revealed that TEN reduced arterial pressure, which was not related to vagus nerve excitation, ganglionic blockade, and peripheral α-adrenergic, M-cholinergic and H1-receptors. At present, there are few studies on the hypotensive effect of PR, and the mechanism of action is not clear, which limits the application of PR in hypotensive. Therefore, further in-depth research is still needed (Peng, 1999).

#### 3.3.2 Lipid-lowering effect

The effects of PR extract (PTE) on lipid accumulation were determined using 3T3-L1 adipocytes and high-fat diet-induced obese mouse models. Next-generation sequencing analysis of liver gene expression and intestinal flora after PTE treatment was performed to elucidate the possible mechanism. It was found that PTE treatment of 3T3-L1 adipocytes could inhibit lipid accumulation in cells by reducing lipid formation and triglyceride content and increasing lipase activity. After 5 weeks of PTE treatment, the weight gain, serum triglyceride content and hepatic steatosis of obese mice induced by high-fat diet were decreased, and the gene expression involved in lipid and cholesterol metabolism was significantly changed. After PTE treatment, low-grade chronic inflammation of obesity caused by high-fat diet also decreased. In addition, PTE treatment improved the relatively low Bacteroidetes/Firmicutes ratio in the intestine of mice fed a high-fat diet by enriching the proteobacteria population and reducing the deferribacteres population. In summary, PTE treatment inhibits lipid accumulation by inducing the expression of the main transcription factor PPARα, reduces low-grade chronic inflammation of obesity, and changes the gut microbiota (Wang C. C. et al., 2017). At present, there are few studies on the hypolipidemic effect of PR. Moreover, this study lacks the evidence of positive control and clinical experimental data. Therefore, the hypolipidemic effect and mechanism of PR still need further study.

#### 3.3.3 Cardiovascular effects

3,4,5-trimethoxycinnamic acid (TMCA), methyl 3,4,5-trimethoxycinnamate (M-TMCA) and p-methoxycinnamic acid (MCA) are the main active metabolites of PR in the treatment of insomnia, anxiety and palpitation. Whole-cell configuration of the patch-clamp technique was used to measure action potential (AP) and membrane currents in single ventricular myocytes enzymatically isolated from adult rabbit hearts. M-TMCA showed antiarrhythmic activity in rabbit ventricular myocytes by shortening the action potential duration during repolarization, inhibiting L-type calcium current, eliminating early afterdepolarization induced by isoproterenol and BayK8644, inhibiting delayed afterdepolarization and triggering activity (Zhao et al., 2013).

TEN has obvious protective effect on myocardial ischemia-reperfusion injury in rats. The mechanism includes inhibiting the increase of CPK in serum and the formation of NO in myocardial tissue, increasing the activity of SOD and reducing the range of myocardial infarction in rats (Guo and Shen, 2005).

### 3.4 Antioxidation effect

Oxidative stress is defined as the imbalance between oxidants (reactive oxygen species/ROS and reactive nitrogen/RNS) and antioxidants. Under the condition of oxidative stress, excessive ROS can destroy cellular proteins, lipids, and DNA, leading to fatal cell damage, which in turn involves a variety of pathology, such as aging, cancer, neurodegenerative diseases, cardiovascular diseases, and diabetes. Therefore, the research and development of traditional Chinese medicine with antioxidant effect has become one of the hotspots of scientific research (Table 9).

**TABLE 9:** Effects of RP on antioxidation.

Pharmacological effects	Extracts/metabolites	Model	Dose range tested	Mechanism	Ref.
Antioxidation effect	Tenuigenin	PC12 cells	5, 10, 20 mg/L	Increase SOD activity and prevent H_2_O_2_-mediated oxidative damage	Sun et al. (2007)
	Tenuigenin	Hippocampal neurons	1, 2, 4 μg/mL	Remove intracellular reactive oxygen species, regulate the activity of Bcl-2 and apoptosis-related proteases	Chen et al. (2010)
	YZ-OE	SAMP mice, SAMR mice	25, 50 mg/kg	Increase the activity of SOD and GSH-Px, reduce the level of MDA	(Liu et al., 2010)
	3,6′-Disinapoyl sucrose	SAMP mice, SAMR mice	25, 50 mg/kg	Increase the activity of SOD and GSH-Px, reduce the level of MDA	Liu et al. (2010)

TEN have significant antioxidant activity, which could reduce LDH leakage, reduce MDA content, increase SOD activity, improve cell damage caused by H_2_O_2_, and increase cell survival rate (Sun et al., 2007). Another study evaluated the effect of TEN on methylglyoxal-induced cell damage in primary cultures of rat hippocampal neurons. MTT and Hoechst 33,342 staining, together with flow cytometric analysis using annexin-V and propidium (PI) label, indicated that TEN also exerted an antioxidant effect in hippocampal neurons by scavenging intracellular reactive oxygen species and regulating the activity of Bcl-2 and apoptosis-related proteases (Chen et al., 2010). After that, the researchers investigated the antioxidant activity of YZ-OE and 3,6′-disinapoyl sucrose in PR. It was found that YZ-OE and 3,6′-disinapoyl sucrose could significantly increase the activity of SOD in serum and glutathione peroxidase (GSH-Px) in hepatocytes, and decrease the level of MDA (Liu et al., 2010). However, this study lacks in-depth research on the antioxidant mechanism, and does not explore the signaling pathways and target proteins related to its antioxidant effect.

### 3.5 Other pharmacological effects

In addition to the above effects, PR also has antitussive, diuretic, inhibition of alcohol absorption and liver protection effects (Table 10).

**TABLE 10:** Other pharmacological effects of PR.

Pharmacological effects	Extracts/metabolites	Model	Dose range tested	Mechanism	Ref.
Anti-sputum and antitussive effects	Tenuigenin	Mice	5, 10, 20 mg/L	—-	Peng and Xu (1998)
Inhibition of alcohol absorption	Tenuigenin	Mice	—-	—-	Yoshikawa et al. (1995)
	Ethanol extract of PR	CCL-13 cells	1–10 μg/mL	Scavenging ROS inhibits mitochondrial-dependent apoptosis pathway	Kim et al. (2019)

PR is usually used to treat cough. Saponin 3D may be the main active metabolites of PR in eliminating phlegm. Saponin 2D and Saponin 3C were the main metabolites of antitussive effect, and the effect was even stronger than that of the same dose of Codeine and Pentoxyverine Citrate Tablets (Peng and Xu, 1998). The saponins in PR could also inhibit the absorption of alcohol in the body to a certain extent., among which senegasaponin A and senegin I were the most significant. The study of structure-activity relationship showed that 28-O-glycosylation and the presence of cinnamoyl substituents are crucial factors (Yoshikawa et al., 1995). Moreover, Alcohol extract of PR has a protective effect on oxidative stress-induced DNA damage and apoptosis of Chang liver cells (Kim et al., 2019).

The pharmacological effects of PR mainly include improving cognitive impairment, improving learning and memory ability, antioxidation, anti-epilepsy, anti-depression, anti-tumor, anti-inflammatory, immune regulation and protecting liver cells. By summarizing the existing research results, it can be seen that the pharmacological effects of PR are mainly based on the central nervous system, and its protective effect on nerves, improvement of cognitive impairment, and improvement of learning and memory ability are particularly prominent. Secondly, the antioxidant, antidepressant and immunomodulatory effects of PR are also clear. The repair of cartilage damage and the protective effect of hepatocytes need to be further studied. In summary, the medicinal value of PR is rich. Further systematic and in-depth research can enrich clinical application ideas, expand research horizons, and will also lay the foundation for the comprehensive development and application of PR.

## 4 Toxic side effects

PR has the traditional effects of treating insomnia, forgetfulness, palpitations, improving intelligence and other neurological symptoms. Saponins are important biologically active metabolites of *Polygala* plants and have neuroprotective effects. Toxicological studies showed that TEN could trigger gastrointestinal toxicity, significantly inhibit gastrointestinal motility, cause gastrointestinal flatulence and intestinal wall thinning, so it needs attention (Table11).

**TABLE 11:** Toxicities and side effects of PR.

Extracts/metabolites	Model	Dose range tested	Mechanism	Ref.
Senegenin	Mice	100, 200 mg/kg	Reducing the gastric PGE2 level	Wen et al. (2015)
Tenuifolin	Mice	100, 200 mg/kg	Reducing the gastric PGE2 level	Wen et al. (2015)
Onjisaponin B	Mice	100, 200 mg/kg	Reducing the gastric PGE2 level	Wen et al. (2015)

The effects of the decoction of PR and its compatibility with *Glycyrrhiza uralensis* Fisch. (Gancao) in different proportions on gastrointestinal motility in mice were observed by small intestinal motility carbon powder propulsion method and gastric emptying colorimetry. The results showed that single raw PR and the compatibility of raw PR and Gancao (3:1) had obvious inhibitory effect on small intestinal motility and gastric emptying in mice, and made gastrointestinal inflation, intestinal wall thinning and necrosis, or even death, showing gastrointestinal toxicity, while the compatibility of PR and Gancao (3:2, 3:3) had no significant effect on gastrointestinal motility. It showed that PR has a certain toxic effect on the gastrointestinal tract (Zhang et al., 2016; He et al., 2023).

Further studies were found that PR could significantly reduce gastric emptying, small intestinal propulsion, and duodenal myoelectric fast and slow wave frequencies in rats, resulting in gastrointestinal electrical wave disorders. TEN is the main toxic substance of PR, which was irritating to the gastrointestinal tract and could cause gastrointestinal motility disorder in rats. The toxicity of TEN was related to the length of the sugar chain in the molecule. Tenuigenin B could significantly increase the contraction amplitude of rabbit isolated intestine and cause gastrointestinal tissue damage in rats (Tian et al., 2005). In 2015, it was found that onjisaponin B (80 mg/L) could lead to irregular and strong contractions in the isolated intestine. At a dose of 200 mg/kg, Onjisaponin B, tenuifolin, or senegenin significantly reduced gastric PGE2 levels, indicating that these saponins may lead to loss of gastric mucosal protection and ultimately gastric damage (Wen et al., 2015). In addition, it has been reported that PR and its total saponins can significantly reduce interstitial cells of Cajal (ICC) in gastric and intestinal myenteric plexus. Therefore, the mechanism by which PR and its total saponins induce gastrointestinal motility may be related to the reduction of pepsin ICC (Wang et al., 2004).

Combined with these experimental results, it could be proved that some metabolites in PR will produce certain toxicological effects. In clinical application, different processing methods of PR and the compatibility ratio with other drugs can effectively reduce the toxicity of PR and enhance the curative effect. At present, the mechanism of gastrointestinal toxicity caused by TEN is not clear, and further research is still needed.

## 5 Conclusions and future perspectives

PR is a kind of Chinese herbal medicine which is widely used in people’s daily life. It has abundant natural resources in China. Clinically, it can be used for palpitations, insomnia, forgetfulness, epilepsy, cough, phlegm, carbuncle, sores, breast swelling and pain. Modern research has provided a comprehensive description of its pharmacological effects, chemical composition and toxicity. More than 160 metabolites have been isolated from PR, mainly including saponins, xanthones, oligosaccharide esters and alkaloids. These metabolites and extracts have a wide range of pharmacological activities, including protective effects on the nervous system, immune system, cardiovascular system, respiratory system, as well as antioxidant, liver protection and other pharmacological activities. In addition, toxicological studies have revealed that improper use of PR can cause toxic reactions, such as sore throat, vomiting, abdominal distension, edema, etc. Tenuigenin are the main metabolites of toxicity and side effects in PR. Therefore, based on the problem that PR has rich pharmacological activity but can cause toxic reactions at the same time, there are several key issues that need to be resolved to further develop PR and improve its clinical application.

First of all, PR is the dry root of *Polyyala tenuifolia* Willd. or *Polygala sibirica* L., but the quality standard of PR in Chinese Pharmacopoeia does not distinguish the interspecific difference between *P. tenuifolia* and *P. sibirica*, nor does it objectively determine their respective quality indicators. The research on medicinal PR is mainly focused on *P. tenuifolia*. There are few systematic studies on *P. sibirica*, and *P. sibirica* has great research potential. Therefore, it is necessary to conduct in-depth research on *P. sibirica*, and to conduct inter-specific comparative studies on *P. tenuifolia* and *P. sibirica* in biology, chemical composition, content determination and pharmacological effects, so as to find out the differences between samples and objectively determine their respective quality indicators. It will provide experimental data for the standardization of Chinese medicine standards, provide scientific basis for ensuring the correct, safe and effective use of drugs by patients, and promote the healthy and orderly development of PR.

Secondly, TEN is not only the main active metabolites of PR with some pharmacological activities such as antitussive, expectorant, intellectual, antihypertensive, and anti-aging, but also a toxic metabolite that can produce gastrointestinal toxicity. Therefore, it is necessary to further explore the balance between the effectiveness and possible toxicity of saponins.

Finally, saponins are the main toxic metabolites of PR. However, the related toxic metabolites and their toxic mechanisms are not yet clear. Clinical studies have shown that processing has a regulatory effect on the quality of PR, which can increase efficiency and reduce toxicity. The processing methods of PR are diverse, mainly Gancao and honey. However, at present, the processing mechanism of related synergistic attenuation has not been fully elucidated, so further research is needed.

In this paper, more than 160 metabolites such as triterpenoid saponins, Xanthones and oligosaccharide esters contained in PR were reviewed, and various pharmacological effects of PR and metabolites were analyzed. As a traditional Chinese medicine with a long history of medication, PR has a significant effect on improving learning and memory ability. It is expected to adopt modern advanced science and technology and methods to carry out in-depth comprehensive research and development and utilization of PR. To explore the role and mechanism of PR and its metabolites in the prevention and treatment of Alzheimer’s disease, and to screen for potential metabolites for the treatment of neurodegenerative diseases, which will help to develop new drugs with definite efficacy and guide clinical further rational compatibility.
